# The Prevalence of Sexual Harassment and Bullying Among Norwegian Afghanistan Veterans: Does Workplace Harassment Disproportionately Impact the Mental Health and Life Satisfaction of Female Soldiers?

**DOI:** 10.1177/08862605241248432

**Published:** 2024-04-30

**Authors:** Line Rønning, Rachel Shor, Frederick Anyan, Odin Hjemdal, Hans Jakob Bøe, Catherine L. Dempsey, Andreas Espetvedt Nordstrand

**Affiliations:** 1 Department of Psychology, Norwegian University of Science and Technology, Trondheim, Norway; 2 Center for the Study of Traumatic Stress, Department of Psychiatry, Uniformed Services University of the Health Sciences, Bethesda, MD, USA; 3 Henry M. Jackson Foundation for the Advancement of Military Medicine, Inc., Bethesda, MD, USA; 4 Institute of Military Psychiatry, Norwegian Armed Forces Joint Medical Services, Oslo, Norway; 5 Department of Psychology, University of Oslo, Oslo, Norway

**Keywords:** sexual harassment, bullying, military veterans, posttraumatic stress disorder, life satisfaction, anxiety, depression

## Abstract

Experiencing sexual harassment and bullying during military service can lead to negative consequences for a soldier’s mental health and life satisfaction, including increased risk of depression, anxiety, and posttraumatic stress. No studies have to date investigated the prevalence and correlates of sexual harassment and bullying among Norwegian Afghanistan veterans, despite the increased global focus on these topics. In 2020, 6,205 Norwegian Afghanistan veterans (8.3% women) completed an online post-deployment survey, including questions about experiences of sexual harassment, bullying, mental health, and life satisfaction. Compared to their male counterparts, female veterans experienced significantly more sexual harassment and bullying during Afghanistan deployment (3.2% vs. 0.04% for experiencing sexual harassment, and 4.0% vs. 1.0% for bullying) and during other military services (14.3% vs. 0.4% for sexual harassment, and 15.9% vs. 3.7% for bullying). Sexual harassment was associated with younger age and experiencing childhood sexual harassment for both women and men, with men also having longer deployments. Bullying was associated with longer deployments and childhood sexual harassment for women, while men who reported bullying more often had longer deployments, held an officer rank, were less inclined to have a spouse/intimate partner, and reported childhood sexual harassment and bullying. Both sexual harassment and bullying were associated with increased risk of mental health problems and reduced life satisfaction for women, but this was only true for bullying among men. Despite lower reported rates of workplace harassment compared to studies from other cultures, this study demonstrates that sexual harassment and bullying in the military can negatively impact soldiers’ mental health and life satisfaction. Notably, female veterans’ mental health and life satisfaction appear to be particularly affected by sexual harassment during military service, an association not seen in males. This underscores the need for gender-specific, cultural, and context-sensitive prevention and support for workplace harassment experiences.

Workplace harassment is a major issue in the armed forces given both its prevalence in many military communities and the detrimental consequences it can have on mental health and life satisfaction (Hendrikx et al., 2021; Kimerling et al., 2010; Moreau et al., 2022; Stuart & Szeszeran, 2021; Wilson, 2018). Moreover, in a military force, experiencing workplace harassment can have an outsized negative impact because it also adversely influences unit cohesion, force readiness, and combat effectiveness, as well as decrease the public esteem of the military among the civilian population (Allard et al., 2011; Klein & Gallus, 2018; Stuart & Szeszeran, 2021). Workplace harassment includes sexual harassment—unwelcome verbal or physical contact of a sexual nature that is hostile in character and interferes with work performance (Department of Defense, 1995), and generalized harassment or bullying—a range of threatening, degrading, and deliberate behavior, characterized by its harmful intent and repetitive nature within a professional environment (Einarsen et al., 2011). Both sexual harassment and bullying are seen as part of a continuum of disruptive and hostile behaviors, with sexual harassment being a specific form of harassment characterized by its sexual nature. Therefore, sexual harassment might be understood as an overlapping construct with bullying.

More than half (52.5%) of female military personnel have experienced workplace sexual harassment—nearly six times more frequently than males (8.9%; Wilson, 2018). Similar high prevalence rates and gender disparities have also been found for workplace bullying (Hendrikx et al., 2021; Magerøy et al., 2009; Ubisch et al., 2023), although these rates are more equivocal (Farmer, 2016). However, these figures are likely underestimations, as incidents of harassment are often unreported (Blais et al., 2018). While a significantly higher proportion of female military personnel report workplace harassment (National Defense Research Institute, 2014; Ubisch et al., 2023), the total number of males experiencing workplace harassment is comparable to that of females, due to the larger number of males in the military. However, this might change, as there is an increasing number of women serving in the armed forces worldwide. For instance, in the Norwegian Armed Forces, the proportion of women serving the initial military service has risen from 5% in 2006 to 36% in 2022 (Meld. St. 36 (2006–2007); Norwegian Armed Forces, 2023).

Beyond knowledge about the prevalence rates of workplace harassment, it is important to comprehend how risk and protective factors can influence the likelihood of experiencing such incidents. Existing studies have found that women, those of younger age, and military personnel in the junior enlisted ranks are more likely to experience workplace harassment (National Defense Research Institute, 2014; Magerøy et al., 2009; Stander & Thomsen, 2016; Stuart & Szeszeran, 2021). Furthermore, another well-established risk factor for workplace harassment for female military personnel and veterans is a prior history of victimization (Williamson et al., 2022; Wilson et al., 2015). Research has demonstrated that experiences of both sexual harassment and bullying are associated with the development of a host of adverse health outcomes, including posttraumatic stress disorder (PTSD), depression, anxiety, substance use disorders, and reduced job and life satisfaction (Campbell-Sills et al., 2023; Hendrikx et al., 2021; Kimerling et al., 2010; Stuart & Szeszeran, 2021; Willness et al., 2007). However, studies exploring gender differences in negative mental health outcomes related to workplace harassment, such as sexual harassment, have yielded inconsistent results. Some studies have found a stronger association between sexual harassment and negative mental health outcomes for women compared to men (Kimerling et al., 2010; Maguen et al., 2012); others have found the opposite (Street et al., 2007; Vogt et al., 2005); while others have found no gender differences (Murdoch et al., 2007). Given that the majority of workplace harassment research to date has focused on female samples (e.g., Burns et al., 2014; Hendrikx et al., 2021; Kelly et al., 2011; Kim et al., 2016; Surìs et al., 2007; Williamson et al., 2022), and considering the substantial number of both men and women in military services worldwide reporting workplace harassment, these findings underscore the need for further investigation into potential gender-specific patterns in prevalence, risk factors, and negative mental health outcomes.

Despite the prevalence and consequences of workplace harassment, no studies have investigated the extent of the mental health impact of experiencing sexual harassment and bullying among Norwegian Afghanistan veterans. There are several recent studies on the topic in other national militaries (e.g., Belgium, the United Kingdom, South Korea, France, and the United States; Buyse et al., 2021; Hendrikx et al., 2021; Kim et al., 2016; Moreau et al., 2022; Wilson, 2018); however, their findings may not be relevant to a Norwegian cohort due to major cultural, operational, and sociodemographic differences. Scandinavian countries, such as Norway, generally have lower levels of reported sexual harassment and bullying in the general population than other comparable nations (León-Pérez et al., 2021; Nielsen et al., 2010). Moreover, rates of mental health problems among military personnel and veterans also vary between different nations and operational contexts (e.g., Hougsnæs et al., 2017; Stevelink et al., 2018). The cultural and context-sensitive nature of sexual harassment and bullying highlights the need for studies specific to the mission and country of origin when researching these phenomena in a particular cohort. For example, one hypothesized mechanism for increased harassment in the military is the prevalence of masculine cultural values. However, these cultural values may not correspond as significantly to Nordic military forces, as research has demonstrated that relative to some other cultures, Nordic countries have been found to hold more egalitarian beliefs on masculine values in the workplace (Estrada & Berggren, 2009).

Given these unique factors that characterize the Nordic context, and thus the Norwegian Armed Forces, the study aimed to fill this research gap. The aim of this study was threefold. First, we aimed to explore the prevalence rates of workplace sexual harassment and bullying among Norwegian Afghanistan veterans. Second, we aimed to explore associated predictors of workplace harassment experiences. Third, we aimed to examine the associations between workplace harassment experiences and subsequent mental health symptoms (PTSD, anxiety, and depression), as well as life satisfaction. Given the known gender differences in rates of workplace harassment (Magerøy et al., 2009; Wilson, 2018), and that men and women experience the risk for mental health disorders following a trauma differently (Kessler et al., 2017), our analysis employed gender-stratified models.

## Method

### Participants and Procedure

The current study used data from a large post-deployment survey of Norwegian veterans following Afghanistan deployment. The study was conducted by the Norwegian Armed Forces Joint Medical Services. All Norwegian military personnel deployed to Afghanistan between 2001 and 2020 were invited to participate in a cross-sectional post-deployment survey (*N* = 9,168) conducted in 2020. Of these, 145 (1.6%) declined participation, and 2,818 (30.7%) did not respond. In total, 6,205 gave their consent to participate in the survey, resulting in a final response rate of 67.7%. The responders (*n* = 6,205) and non-responders (*n* = 2,963) of the survey were compared on selected demographic characteristics to explore the representativeness of the sample (see Supplemental material Table 1). Sample size variations due to missing demographic data and missing responses are reflected in the *n* values associated with specific analyses.

Information about the study was sent to all potential participants, followed by a text message with a link to the web-based survey. The data collection took place between September and November 2020 and included two reminders for those who did not respond. As an incentive to participate, everyone who responded to the survey was included in a lottery for 30 tablets.

The participants in the 2020 Afghanistan Survey filled out a questionnaire on mental and physical health, life satisfaction, and experiences of potentially traumatic incidents experienced both during deployment and outside military service. The survey responses were then linked with the Norwegian Armed Forces Health Registry’s’ administrative records. All procedures, data collection, storage, and distribution of data were made in accordance with the legislation regulating the Norwegian Armed Forces Health Registry. The study was approved by the Regional Committee for Medical and Health Research Ethics of South-East Norway (case number: 33032).

### Variables and Instruments

#### Sexual Harassment and Bullying

The respondents were asked whether they had experienced sexual harassment (*Have you ever been exposed to unwelcomed sexual conduct?)* or bullying/harassment (*Have you ever been bullied/harassed?)* during deployment to either Afghanistan and/or in another military setting. Furthermore, participants provided information on whether they had experienced sexual harassment and/or bullying before 18 years of age, which was referred to as *Childhood sexual harassment* and *Childhood bullying* for the current study.

#### Mental Health and Global Life Satisfaction

Past week posttraumatic stress symptoms were measured with the 10-item Posttraumatic Symptom Scale (Holen et al., 1983). The items were rated on a Likert scale from 1 (*never/rarely*) to 7 (*very often*), giving a possible score range of 10 to 70 (α = .91). Current symptoms of anxiety and depression were captured with the 14-item Hospital Anxiety and Depression Scale (Zigmond & Snaith, 1983). The items were rated on a scale ranging from 0 to 3, giving a total score range of 0 to 21 for both anxiety (α = .81) and depression (α = .83), with higher scores indicating higher symptom severity. Lastly, global life satisfaction was measured with the five-item Satisfaction with Life Scale (Diener et al., 1985; Pavot & Diener, 1993) The items were rated on a scale ranging from 1 (*Strongly Disagree*) to 7 (*Strongly Agree*), giving a total score range of 5 to 35 (*α* = .92), with higher scores indicating greater life satisfaction.

#### Sociodemographic and Military Characteristics

Sociodemographic and military service information was obtained through the administrative records of the Norwegian Armed Forces Health Registry in 2020 and included information about a number of international deployments, time since the last international deployment, total deployment length, and military rank the participants had at their last international deployment. The Norwegian military and civilian rank system is equivalent to the North Atlantic Treaty Organization (NATO) system and is in this article divided into civilian ranks, enlisted ranks (OR1–OR4), non-commissioned officers (OR5–OR9), junior officers (OF1–OF2), and senior officers (OF3–OF9). Participants provided further information about exposure to war zone traumatic experiences during the Afghanistan deployment, highest educational attainment, and whether they had a spouse/intimate partner when the survey was conducted.

### Data Analysis

Descriptive analyses were used to compare the demographic and military service characteristics of women and men in the sample. To assess gender differences in the frequency of sexual harassment and bullying, we performed chi-square tests. Furthermore, to examine variables associated with experiencing sexual harassment or bullying during military service, we utilized gender-stratified multivariate logistic regression. Age, having a spouse or intimate partner, international deployment length, rank, childhood sexual harassment, and childhood bullying were included as independent variables.

Finally, we used linear regressions to examine the associations of symptom severity (PTSD, depression, and anxiety) and global life satisfaction, with sexual harassment and bullying. Age, having a spouse or intimate partner, total international deployment time, rank, time since deployment, war zone exposure, and childhood sexual harassment and bullying were included in all regressions to control for potential confounding effects of these variables on the associations of interest. Total international deployment time was included in all analyses as it captures the strain related to being absent from family and civilian social life, the burden on intimate relationships, physical constraints and workload, and the time of potential separation from spouse or intimate partner. Childhood sexual harassment and bullying were also included in the analysis to control for potential cumulative or retraumatizing experiences of later sexual harassment and/or bullying. We ran each analysis separately for men and women to identify gender-specific patterns in the association between workplace harassment and mental health and global life satisfaction.

Due to non-normality, the bootstrapping method with 1,000 replications was applied to estimate a bias-corrected 95% confidence interval (CI). As the proportion of missing data on all variables was less than 5%, missing data were not replaced (Schafer, 1999). P-values less than or equal to 0.05 were regarded as statistically significant. All statistical analyses were conducted in Stata (v17; StataCorp, 2021).

## Results

### Description of the Study Population

The demographic characteristics of the sample are shown in Table 1. As compared to men, women had significantly shorter deployments; less deployments; shorter time had elapsed since their last deployment; were less inclined to have a spouse or intimate partner; and had less overall war zone trauma exposure; were less inclined to hold enlisted ranks, but more inclined to hold civilian or junior officer ranks. Over half of the women (58.8%) had at least a master’s degree compared to one-third of the males.

**Table 1.** Characteristics of the Study Sample, by Gender.

Variable	Women				Men				
	*n*	%	*M*	*SD*	*n*	%	*M*	*SD*	*p*
Age (years)	512		42.0	10.0	5,693		41.9	9.5	.846
Number of international deployments	510		4.8	3.9	5,678		5.8	4.3	<.001
Total international deployment length (months)	510		10.9	9.1	5,678		14.4	12.1	<.001
Time since last international deployment (years)	510		9.1	4.3	5,678		9.7	4.4	<.01
Afghanistan deployment length (months)	510		7.5	4.4	5,669		8.7	5.5	<.001
Last rank									
Civilian	25	5.1			95	1.7			<.001
Enlisted	73	14.9			1,589	28.5			<.001
Non-commissioned officer	37	7.5			552	9.9			.092
Junior officer	251	51.1			2,140	38.3			<.001
Senior officer	105	21.4			1,208	21.6			.898
With a spouse/partner	357	69.7			4,602	81.0			<.001
Educational attainment									
Primary school	1	0.2			71	1.3			.029
Secondary school	26	5.1			860	15.1			<.001
Vocational training	15	2.9			814	14.3			<.001
University, lower degree	169	33.0			2,063	36.3			.136
University, higher degree	301	58.8			1,873	33.0			<.001
War zone exposure load									
None	56	11.0			394	7.0			.001
Low	240	47.1			1,901	33.7			<.001
Medium	144	28.2			1,598	28.3			.976
High	70	13.7			1,754	31.1			<.001
Workplace harassment									
Sexual harassment	71	14.3			20	0.4			<.001
Bullying	79	15.9			199	3.7			<.001
Anxiety symptoms	493		3.6	3.2	5,433		3.4	3.2	.135
Depression symptoms	493		2.1	2.9	5,433		2.5	3.1	<.05
Posttraumatic stress symptoms	495		16.9	8.4	5,437		16.0	8.3	<.05
Life satisfaction	493		28.6	5.8	5,414		28.0	6.1	<.05

*Note*. Some columns do not add up to the column total due to missing data. *SD* = standard deviation.

### Prevalence of Sexual Harassment and Bullying

Sexual harassment during any military service was reported by 14.3% of the women and 0.4% of the men (odds ratio [OR] = 45.06, 95% CI [26.77, 78.78], *p* < .001). Of those reporting sexual harassment during military service, 3.2% of the women and 0.04% of the men reported experiencing it during Afghanistan deployment (OR = 90.26, 95% CI [21.08, 809.89], *p* < .001). Experiencing bullying during military service was reported by 15.9% of the women and 3.7% of the men (OR = 4.98, 95% CI [3.71, 6.62], *p* < .001), of which 4.0% of the women and 1.0% of the men reported experiencing it during Afghanistan deployment (OR = 4.34, 95% CI [2.43, 7.47], *p* < .001). A smaller but meaningful contingent reported experiencing both types of workplace harassment during any military service, with 6.5% of women and 0.04% of men reporting sexual harassment and bullying (OR = 190.04, 95% CI [47.35, 1650.87], *p* < .001). Moreover, the analysis revealed a significant association between experiencing sexual harassment and experiencing bullying (χ^2^(1, *N* = 5,937) = 153.03, *p* < .001). Childhood sexual harassment was reported by 12.7% of the women and 3.2% of the men (OR = 4.41, 95% CI [3.20, 6.02], *p* < .001). Lastly, childhood bullying was reported by 21.9% of the women and 26.3% of the men (OR = 0.79, 95% CI [0.62, 0.98], *p* < .05).

### Predictors of In-Service Sexual Harassment and Bullying

Women who experienced sexual harassment during military service were younger and had experienced childhood sexual harassment. Men who experienced sexual harassment during military service were younger, had longer international deployments, and had experienced childhood sexual harassment, as shown in Table 2. Women who had experienced bullying had longer international deployments, and experienced childhood sexual harassment. Men who had experienced bullying had longer international deployments, held an officer rank, were less likely to have a spouse or intimate partner, experienced childhood sexual harassment, and childhood bullying (Table 2).

**Table 2.** Sociodemographic and Military Characteristics Associated with Sexual Harassment and Bullying, by Gender.

Variables	Sexual Harassment		Bullying	
	aOR [95% CI]	*p*	aOR [95% CI]	*p*
Women (*n* = 476)				
Age	0.96 [0.93, 0.99]	.005	0.97 [0.94, 1.00]	.080
Total international deployment length (months)	1.01 [0.98, 1.05]	.484	1.01 [1.00, 1.03]	.017
Rank (officer)	0.85 [0.46, 1.58]	.611	1.67 [0.81, 3.44]	.164
Spouse/intimate partner	0.99 [0.55, 1.77]	.963	0.61 [0.34, 1.07]	.084
Childhood sexual harassment	2.13 [1.00, 4.56]	.050	2.33 [1.19, 4.57]	.014
Childhood bullying	0.71 [0.35, 1.45]	.348	1.63 [0.91, 2.93]	.101
Men (*n* = 5,336)				
Age	0.93 [0.86, 0.99]	.041	1.00 [0.98, 1.01]	.622
Total international deployment length (months)	1.03 [1.00, 1.06]	.011	1.01 [1.00, 1.02]	.021
Rank (officer)	2.41 [0.71, 8.16]	.156	1.85 [1.38, 2.83]	.000
Spouse/intimate partner	2.56 [0.78, 8.45]	.122	0.62 [0.45, 0.86]	.004
Childhood sexual harassment	5.20 [1.48, 18.34]	.010	3.63 [2.17, 6.08]	.000
Childhood bullying	2.14 [0.83, 5.51]	.115	1.87 [1.36, 2.57]	.000

*Note*. aOR = adjusted odds ratio.

The dichotomous rank categorization (officer ranks vs. other ranks) did not predict sexual harassment for either men or women but was a significant predictor for bullying among males. As such, more fine-grained differences between ranks may have an importance. We therefore conducted a series of post hoc analyses with chi-square contingency tables, using Bonferroni corrections to adjust for multiple comparisons. No differences between ranks emerged for women (see Supplemental material Table 2). However, male junior officers were more likely to report experiences of bullying (*χ*^2^[1, *N* = 5,336] = 9.13, *p* < .01) relative to all other military ranks, and enlisted males were less likely to report bullying relative to all other ranks combined (χ^2^[1, *N* = 5,336] = 13.90, *p* < .001).

### Mental Health Symptoms and Global Life Satisfaction

Both sexual harassment and bullying were significant predictors for global life satisfaction, controlling for all the relevant predictors for women (Table 3) indicating as the level of sexual harassment and bullying increases, the global life satisfaction score decreases. For males, experiencing in-service bullying was a significant predictor for global life satisfaction, after controlling for other variables (Table 4). For women, both sexual harassment and bullying were significant predictors for PTSD symptoms, anxiety symptoms, and depression symptoms, indicating that as the level of sexual harassment and bullying increases, the symptom scores increase (Table 3). For males, experiencing bullying was a significant predictor for PTSD symptoms, anxiety, and depression symptoms for men, after controlling for other predictors (Table 4).

**Table 3.** For Women: Linear Regression of Risk and Protective Factors Associated with Mental Health Morbidity.

Variables	Global Life Satisfaction (*n* = 472)			PTSD (*n* = 474)			Anxiety (*n* = 472)			Depression (*n* = 472)		
	*B*	*SE*	*p*	*B*	*SE*	*p*	*B*	*SE*	*p*	*B*	*SE*	*p*
Age	–0.03	0.03	.285	0.02	0.05	.710	–0.02	0.02	.204	0.01	0.02	.641
Having a spouse or intimate partner	2.75	0.60	.000	–0.51	0.82	.529	0.15	0.30	.624	–0.08	0.30	.799
Military variables												
Total international deployment length (months)	0.009	0.03	.799	–0.02	0.04	.699	–0.02	0.02	.164	–0.02	0.02	.284
Rank (officer)	–0.07	0.59	.902	–0.38	0.87	.667	–0.11	0.35	.765	0.12	0.31	.687
Time since last deployment (years)	–0.04	0.06	.546	0.19	0.09	.042	0.07	0.04	.043	0.04	0.03	.193
Trauma exposure type												
Sexual harassment	–3.12	0.85	.000	4.77	1.18	.000	1.49	0.44	.001	1.23	0.43	.004
Bullying	–1.98	0.82	.016	4.56	1.28	.000	1.66	0.42	.000	1.54	0.45	.001
War zone exposure	–0.11	0.05	.017	0.29	0.08	.000	0.12	0.03	.000	0.09	0.03	.000
Childhood sexual harassment	–1.04	0.81	.195	1.99	1.23	.105	0.95	0.44	.032	0.30	0.42	.475
Childhood bullying	–1.52	0.64	.018	–0.18	0.82	.830	0.35	0.33	.289	0.53	0.31	.086

*Note.* PTSD = posttraumatic stress disorder; *SE* = standard error.

**Table 4.** For Men: Linear Regression of Risk and Protective Factors Associated with Mental Health Morbidity.

Variables	Global Life Satisfaction (*n* = 5,311)			PTSD (*n* = 5,333)			Anxiety (*n* = 5,329)			Depression (*n* = 5,329)		
	*B*	*SE*	*p*	*B*	*SE*	*p*	*B*	*SE*	*p*	*B*	*SE*	*p*
Age	0.01	0.01	.147	–0.02	0.01	.150	–0.03	0.01	.000	–0.02	0.004	.000
Having a spouse or intimate partner	4.24	0.25	.000	–2.27	0.35	.000	–0.55	0.13	.000	–0.93	0.13	.000
Military variables												
Total international deployment length (months)	–0.03	0.01	.000	0.02	0.01	.075	–0.003	0.004	.393	0.01	0.004	.002
Rank (officer)	1.38	0.19	.000	–1.56	0.26	.000	–0.47	0.10	.000	–0.35	0.10	.000
Time since last deployment (years)	–0.08	0.02	.000	0.16	0.03	.000	0.07	0.01	.000	0.05	0.01	.000
Trauma exposure type												
Sexual harassment	–1.28	1.38	.353	0.35	2.18	.874	0.74	0.83	.374	–0.26	0.66	.696
Bullying	–3.43	0.51	.000	4.64	0.74	.000	1.73	0.27	.000	1.76	0.27	.000
War zone exposure	–0.07	0.01	.000	0.23	0.02	.000	0.07	0.01	.000	0.07	0.01	.000
Childhood sexual harassment	–1.36	0.53	.010	2.89	0.68	.000	1.16	0.27	.000	0.64	0.27	.017
Childhood bullying	–1.72	0.19	.000	2.17	0.27	.000	0.70	0.10	.000	0.71	0.10	.000

*Note.* PTSD = posttraumatic stress disorder; *SE* = standard error.

## Discussion

### Gender Differences in Experiencing Sexual Harassment and Bullying

Using a large sample of female and male veterans, the current study represents the first comprehensive investigation on the scope of sexual harassment and bullying among Norwegian Afghanistan veterans. As expected, this study demonstrated that there is a meaningful gender gap in self-reported experiences of sexual harassment and bullying during deployment. In line with many findings among female soldiers of different nationalities (Hendrikx et al., 2021; Kimerling et al., 2010; Moreau et al., 2022; Stuart & Szeszeran, 2021; Wilson, 2018), Norwegian women who served in Afghanistan were significantly more likely than men to report experiences of sexual harassment and bullying. The military profession, at its core, is about the systematic practice of violence (Kümmel, 2002), an activity traditionally dominated by masculine ideals such as toughness, aggression, violence, control, courage, and domination (Eichler, 2014). In Norway, as in most militaries around the world, the majority of the employees in the Norwegian Armed Forces are men (Norwegian Armed Forces, 2023). Accordingly, a skewed gender ratio combined with the fact that military work tasks require “traditional” masculine qualities can increase the risk of experiencing sexual harassment and bullying for women in the military (Kümmel, 2002; Nielsen & Einarsen, 2018; Willness et al., 2007). This is consistent with the hypothesis that perpetrators of victimization may be more likely to target individuals with less sociocultural power, as they may be less likely to fight back or to report victimization because of concerns about disbelief or fear of ostracism and sanctions related to hierarchy, group cohesion, and gender stereotypes (Allard et al., 2011; Bell et al., 2018; McCone et al., 2018). Correspondingly, females reported significantly more sexual harassment and bullying than males, in addition to younger age was associated with sexual harassment for both genders. However, contrary to the sociocultural hypothesis, we found that holding junior officer ranks was a significant predictor for experiencing bullying for men, whereas enlisted males were less likely to report bullying than the other ranks. Research has shown that those having little formal and informal power (i.e., holding junior ranks and those who are younger) report more bullying than senior officers (Farmer, 2016; Magerøy et al., 2009). However, this does not explain why junior officers reported more, and enlisted males reported significantly less bullying. One hypothesis is that men holding medium power positions (junior officers) may be at risk of experiencing bullying from seniors, but also from those holding lower ranks, as they become middle managers and may have to bridge the gap between lower and higher ranks. Another hypothesis is that there might exist reporting or perception bias, as the enlisted males may be less likely to recognize or report incidents of bullying, due to social norms, fear of reprisal from higher ranked military personnel, or fear of retaliation or impact on career (National Defense Research Institute, 2014). Yet, there remains a need to further explore the prevalence of sexual harassment and bullying across ranks, to develop an understanding of who is being victimized.

The prevalence rates of sexual harassment experienced during Afghanistan deployment in our study were surprisingly low in comparison to a domestic report (Ubisch et al., 2023), and international studies on the topic (Buyse et al., 2021; Wilson, 2018). For example, the domestic report found that 45% of females and 14% of males had experienced sexual harassment (Ubisch et al., 2023). In addition, a U.S. meta-analysis revealed that an average of 52.5% of females and 8.9% of males had experienced sexual harassment (Wilson, 2018), whereas a study within the Belgian Armed Forces found that over their careers, 67.4% of women reported instances of sexual harassment (Buyse et al., 2021). Differences regarding domestic prevalence rates might be related to the fact that the study sample was deployed internationally, whereas Ubisch et al. (2023) investigated sexual harassment and bullying among all employees and students in the Norwegian Armed Forces. Previous research has demonstrated that deployed males had a lower risk of experiencing military sexual trauma compared to non-deployed males (Barth et al., 2016), which is in line with our findings, although these findings are not consistent across studies (LeardMann et al., 2013). A possible explanation for our findings is that the Afghanistan veterans represent a particularly well selected, trained, and professional segment of the Norwegian Armed Forces. Deployment to a combat-intensive theater such as Afghanistan involves a substantial increase in threat level compared to domestic service, therefore putting high demands on professionalism and mission focus both at the individual level and in how to interact with fellow soldiers. Professionalism tends to foster a sense of mutual morale, engagement, cohesion, and teamwork among deployed military personnel, and may thus protect or prevent sexual harassment and bullying (Klein & Gallus, 2018; Thomsen et al., 2018). Moreover, the Norwegian Armed Forces use a rigorous selection process for international operations, emphasizing favorable health characteristics, in addition to international deployment being voluntary, leading to self-selection where individuals apply if they feel capable and motivated. As a result, this might filter out individuals with a heightened risk of sexually harassing or bullying others, or those with a vulnerability to developing mental health problems as compared to other nations with less extensive selection procedures.

While Anglo cultures (United States, Canada, Great Britain) and Nordic cultures (Norway, Sweden, Denmark) share many similarities, Nordic cultures hold more egalitarian beliefs on masculine values in the workplace than for example, the United States (Estrada & Berggren, 2009). Furthermore, in Norway, women have had the option to serve in the military voluntarily since 1985, granting them equal access to all military positions and training, including combat, alongside their male counterparts. Two decades later, in 2015, Norway expanded mandatory military service to include women (Meld. St. 36 (2006–2007)), increasing the number of women serving in the Armed Forces (Norwegian Armed Forces, 2023). Women are no longer an underrepresented minority in the Norwegian Armed Forces, as they comprise 20% of all employed personnel and 36% of the soldiers in the initial military service (Norwegian Armed Forces, 2023). The relatively less skewed gender ratio in the Norwegian Armed Forces, coupled with egalitarian beliefs within the culture, may contribute to a lower incidence of workplace harassment compared to non-Norwegian or Nordic countries.

### Mental Health Problems and Global Life Satisfaction

Surprisingly, we did not find sexual harassment to have a significant association with mental health problems or life satisfaction for men. This goes against previous studies, which found military sexual trauma and interpersonal violence to be associated with mental health problems for both genders (Iverson et al., 2013; Kimerling et al., 2010; Surìs et al., 2007). However, the lack of significant findings may be due to a low sample size (Murdoch et al., 2006), as such, we cannot exclude an association between sexual harassment and mental health morbidity and reduced life satisfaction among men. Another explanation is that experiences of sexual harassment can be associated with particularly high social stigma for male victims, which may increase barriers to disclose their experiences to others or prompt minimalizing of the occurred event’s severity and intent (e.g., joking the incident away and labeling it as misplaced humor; Bell et al., 2014; Turchik & Wilson, 2010). Accordingly, fear of stigma might have led men in our sample to either minimalize their experiences of sexual harassment or not report them at all, which again makes it difficult to explore the impact of sexual harassment on mental health symptoms and life satisfaction.

The lack of impact from sexual harassment on life satisfaction and mental health symptomology for the male veterans in our sample is, however, in line with previous studies that find women to be more at risk of mental health problems following interpersonal trauma than men (Lilly & Valdez, 2012). In the current study, we replicate such results, by finding sexual harassment to be significantly associated with adverse outcomes in female veterans, but not in male veterans.

Importantly, juxtaposed with reports of global life satisfaction (Pavot & Diener, 1993) and mental health in civilian cohorts (Hinz & Brähler, 2011; Stoll et al., 1999), our sample of Norwegian Afghanistan veterans reported relatively high life satisfaction and low levels of mental health complaints. This underscores the hypothesis that a comprehensive selection process not only promotes professionalism but may also exclude individuals with a higher susceptibility to developing mental health issues, compared to other nations with less rigorous selection procedures.

### Limitations

The findings presented in this study should be interpreted within the context of some limitations. First, the data were self-reported and retrospective which might have led to recall bias and systematic response distortions in the reported findings. Second, the study’s cross-sectional nature precludes that any causal relationships can be inferred, and we were unable to ascertain the recency of the experiences of workplace harassment relative to the current report of mental health symptoms and life satisfaction. Third, many subgroup analyses were conducted with a small number of participants, leading to insufficient power to detect possible differences, and making them vulnerable to errors. Lastly, the study did not define or operationalize sexual harassment and bullying experiences for the participants. This lack of definitions might have led to respondents misinterpreting the terms, potentially affecting the accuracy and understanding of the reported experiences. Consequently, the limitations in the measurement of these variables might have caused participants to report the same incident as both sexual harassment and bullying. Future research should address this issue and develop a standardized questionnaire that investigates different types of bullying and sexual harassment in military populations, as different subtypes of workplace harassment experiences may impose varying risks of mental health problems. Furthermore, only two types of violence were explored, and inquiring about other types of violence (e.g., verbal abuse and physical violence) would have given a more comprehensive picture of the type and prevalence of workplace violence.

Of note, the non-responder analysis revealed significant differences regarding gender (overrepresentation of women), age, and duration of deployment, suggesting that further research is needed to explore these differences to better understand the factors contributing to non-responsiveness, and how to ensure generalizability of results. Although these findings are similar to recent studies in Europe (Hendrikx et al., 2021; Moreau et al., 2022), one should be cautious in generalizing the current findings to other military cultures, for example, the U.S. Armed Forces.

## Conclusion

This is the first study to comprehensively investigate the scope and consequences of experiencing sexual harassment and bullying among Norwegian Afghanistan veterans. The findings suggest that among female veterans, one in six experienced bullying, and one in seven reported having experienced sexual harassment during their military service, whereas the rates for men were substantially lower. Moreover, the prevalence rates for experiencing sexual harassment and bullying during Afghanistan deployment were considerably lower for both women and men (3.2% vs. 0.04% for sexual harassment, and 4% vs. 1% for bullying).

While this study acknowledges lower reported rates of workplace harassment compared to other cultural studies, it underscores the negative impact of sexual harassment and bullying in the military on soldiers’ mental health and life satisfaction. It is particularly noteworthy that female veterans’ mental health and life satisfaction seem to be disproportionately affected by sexual harassment during military service, an association not found among males. This finding emphasizes the need for prevention and support measures that are tailored to gender, culture, and context when addressing workplace harassment experiences.

## Supplemental Material

sj-docx-1-jiv-10.1177_08862605241248432 – Supplemental material for The Prevalence of Sexual Harassment and Bullying Among Norwegian Afghanistan Veterans: Does Workplace Harassment Disproportionately Impact the Mental Health and Life Satisfaction of Female Soldiers? Supplemental material, sj-docx-1-jiv-10.1177_08862605241248432 for The Prevalence of Sexual Harassment and Bullying Among Norwegian Afghanistan Veterans: Does Workplace Harassment Disproportionately Impact the Mental Health and Life Satisfaction of Female Soldiers? by Line Rønning, Rachel Shor, Frederick Anyan, Odin Hjemdal, Hans Jakob Bøe, Catherine L. Dempsey and Andreas Espetvedt Nordstrand in Journal of Interpersonal Violence
