# Supplementary material for: The Prevalence of Sexual Harassment and Bullying Among Norwegian Afghanistan Veterans: Does Workplace Harassment Disproportionately Impact the Mental Health and Life Satisfaction of Female Soldiers?
Source: J Interpers Violence. 2024 Apr 30;40(1-2):465–88. doi: 10.1177/08862605241248432 (PMC11538798; doi:10.1177/08862605241248432)
Supplement: sj-docx-1-jiv-10.1177_08862605241248432 – Supplemental material for The Prevalence of Sexual Harassment and Bullying Among Norwegian Afghanistan Veterans: Does Workplace Harassment Disproportionately Impact the Mental Health and Life Satisfaction of Female Soldiers? [file sj-docx-1-jiv-10.1177_08862605241248432.docx]

**Supplementary materials**

| **Supplementary Table 1.** Comparisons of Responders and Non-Responders | | | | | | | | | |
| --- | --- | --- | --- | --- | --- | --- | --- | --- | --- |
|  | Total |  | Responders | |  | Non-Responders | |  |  |
| Variables | *N* |  | *n* |  |  | *n* |  |  | *p* |
| Gender  Male  Female | 9168 |  | 5693  512 | 91.7%  8.3% |  | 2773  190 | 93.6%  6.4% |  | < .01 |
| Age group (years)  ≤ 29  30-39  40-49  50-59  ≥ 60 | 9167 |  | 222  2844  1739  1064  336 | 3.6%  45.8%  28.0%  17.1%  5.4% |  | 184  1322  892  449  115 | 6.2%  44.6%  30.1%  15.2%  3.9% |  | < .001 |
| Age (mean, SD) | 9167 |  | 41.9 | 9.5 |  | 41.0 | 9.2 |  | < .001 |
| Years since last Afghanistan deployment  0-4  5-9  10-14  ≥ 15 | 9016 |  | 407  2448  2560  754 | 6.6%  39.7%  41.5%  12.2% |  | 215  1103  1157  372 | 7.6%  38.7%  40.6%  13.1% |  | .215 |
| Duration (months) of Afghanistan deployments (mean, SD) | 9086 |  | 8.6 | 5.5 |  | 8.0 | 5.4 |  | < .001 |
| Number of Afghanistan deployments (mean, SD) | 9086 |  | 4.2 | 3.2 |  | 4.1 | 3.5 |  | .286 |
| Number of international deployments (mean, SD) | 9124 |  | 5.7 | 4.3 |  | 5.6 | 4.5 |  | .160 |
| Age at first international deployment (mean, SD) | 9123 |  | 26.9 | 7.7 |  | 26.6 | 7.2 |  | .107 |

| **Supplemental Table 2.** Percentage of Women and Men Reporting Sexual Harassment and Bullying Across Military Ranks | | | | | | | | | | | |
| --- | --- | --- | --- | --- | --- | --- | --- | --- | --- | --- | --- |
|  | Sexual Harassment | | | | |  | Bullying | | | | |
|  | Women (*n* = 477) | |  | Men (*n* = 5336) | |  | Women (*n* = 476) | |  | Men (*n* = 5336) | |
| Rank | *n/total in group* | % |  | *n/total in group* | *%* |  | *n/total in group* | % |  | n*/total in group* | % |
| Civilian | 4 /25 | 16.0% |  | 0/91 | 0% |  | 1/25 | 4.0% |  | 3/91 | 3.3% |
| Enlisted | 12/69 | 17.4% |  | 5/1491 | 0.3% |  | 8/69 | 11.6% |  | 32/1491 | 2.1%** |
| Non-commissioned officer | 9/36 | 25.0% |  | 1/526 | 0.2% |  | 7/36 | 19.4% |  | 18/526 | 3.4% |
| Junior officer | 33/244 | 13.5% |  | 13/2052 | 0.6% |  | 40/243 | 16.5% |  | 96/2052 | 4.7%* |
| Senior officer | 13/103 | 12.6% |  | 1/1176 | 0.1% |  | 19/103 | 18.4% |  | 48/1176 | 4.1% |
| Total | 71/477 | 14.9% |  | 20/5336 | 0.4% |  | 75/476 | 15.7% |  | 197/5336 | 3.7% |
| *Note*. Each one of the ranks are compared to the four other ranks combined.  Information about rank is missing from 36 female and 357 male participants. Missing data was handled with pairwise deletion, and was not replaced due to low missingness (<10%; Bennett, D. A. (2001). How can I deal with missing data in my study? *Australian and New Zealand journal of public health*, *25*(5), 464-469.).  * *p*< .01, ** *p*< .001. | | | | | | | | | | | |
